# Titanium Dioxide Presents a Different Profile in Dextran Sodium Sulphate-Induced Experimental Colitis in Mice Lacking the IBD Risk Gene Ptpn2 in Myeloid Cells

**DOI:** 10.3390/ijms22020772

**Published:** 2021-01-14

**Authors:** Javier Conde, Marlene Schwarzfischer, Egle Katkeviciute, Janine Häfliger, Anna Niechcial, Nathalie Brillant, Roberto Manzini, Katharina Bäbler, Kirstin Atrott, Silvia Lang, Michael Scharl

**Affiliations:** Department of Gastroenterology and Hepatology, University Hospital Zurich, University of Zurich, 8091 Zurich, Switzerland; marlene.schwarzfischer@usz.ch (M.S.); egle.katkeviciute@usz.ch (E.K.); janine.haefliger@usz.ch (J.H.); anna.niechcial@usz.ch (A.N.); natandreu@gmail.com (N.B.); roberto.manzini@usz.ch (R.M.); katharina.baebler@gmail.com (K.B.); kirstin.atrott@usz.ch (K.A.); silvia.lang@usz.ch (S.L.)

**Keywords:** titanium dioxide, ulcerative colitis, macrophages, DSS colitis

## Abstract

Environmental and genetic factors have been demonstrated to contribute to the development of inflammatory bowel disease (IBD). Recent studies suggested that the food additive; titanium dioxide (TiO_2_) might play a causative role in the disease. Therefore, in the present study we aimed to explore the interaction between the food additive TiO_2_ and the well-characterized IBD risk gene protein tyrosine phosphatase non-receptor type 2 (*Ptpn2*) and their role in the development of intestinal inflammation. Dextran sodium sulphate (DSS)-induced acute colitis was performed in mice lacking the expression of *Ptpn2* in myeloid cells (*Ptpn2^LysMCre^*) or their wild type littermates (*Ptpn2^fl/fl^*) and exposed to the microparticle TiO_2_. The impact of *Ptpn2* on TiO_2_ signalling pathways and TiO_2_-induced IL-1β and IL-10 levels were studied using bone marrow-derived macrophages (BMDMs). *Ptpn2^LysMCre^* exposed to TiO_2_ exhibited more severe intestinal inflammation than their wild type counterparts. This effect was likely due to the impact of TiO_2_ on the differentiation of intestinal macrophages, suppressing the number of anti-inflammatory macrophages in *Ptpn2* deficient mice. Moreover, we also found that TiO_2_ was able to induce the secretion of IL-1β via mitogen-activated proteins kinases (MAPKs) and to repress the expression of IL-10 in bone marrow-derived macrophages via MAPK-independent pathways. This is the first evidence of the cooperation between the genetic risk factor *Ptpn2* and the environmental factor TiO_2_ in the regulation of intestinal inflammation. The results presented here suggest that the ingestion of certain industrial compounds should be taken into account, especially in individuals with increased genetic risk

## 1. Introduction

General hypothesis suggests that an uncontrolled inflammatory response, most likely driven by the microbiome and a defective intestinal barrier function, promote a vicious cycle that leads to chronic intestinal inflammation [1,2]. This aberrant immune response is likely driven by genetic variations in the susceptible host [3,4,5]. Increasing evidence also suggests environmental triggers as critically contributors to the pathogenesis of inflammatory bowel disease (IBD) [6]. This theory is supported by the observed increase in IBD incidence in developed countries after the second half of the 20th century [7], together with the rising incidence detected over the last decades in newly industrialized countries, which are adopting a Western lifestyle [8]. 

Among these environmental factors, dietary components and particularly, inorganic nanoparticles and microparticles, which are used as food additives to manipulate food colour and consistency, have gained special attention in IBD research [6,9]. Some of the most widely applied nano-/microparticles used as a food additive are the sub-micron sized (0.1–100 μm) inorganic compounds of titanium dioxide (TiO_2_, also known as E171). There are numerous dietary sources of TiO_2_, e.g., sweets, toothpaste, flour, etc. The total daily intake of TiO_2_ varies among the population, but it is estimated to be 1.28 mg/kg bodyweight [10], suggesting that this microparticle is ingested in substantial amounts on a daily basis. Moreover, it can accumulate in the digestive system and other tissues in mouse and human [11,12,13], therefore the dietary exposure of TiO_2_ should be factored in when addressing the pathogenesis of intestinal inflammation. 

In fact, administration of TiO_2_ microparticles to mice subjected to dextran sodium sulphate (DSS)-induced colitis has been shown to aggravate colon inflammation through the regulation of the Nlrp3 inflammasome [14]. In the same way, mice subjected to long-term administration of TiO_2_ showed a lower body weight and increased signs of colon inflammation, even without DSS treatment [15]. Also, it has been shown that TiO_2_ increased the tumour burden in the inflammation-associated azoxymethane/dextran sodium sulphate (AOM/DSS) model of tumorigenesis [16]. Interestingly, this effect was accompanied by a reduction in goblet cell numbers and an enhanced activation of the pro-inflammatory NFκB signalling pathway [16].

Since single nucleotide polymorphisms (SNPs) in more than 240 gene loci have been associated with an altered susceptibility for IBD pathogenesis [3,5,17,18], genetic predisposition is also considered a relevant risk factor for the disease. These genetic variations have been identified, among others, in members of the family of non-receptor type protein tyrosine phosphatases (PTPNs) [5,19,20]. Particularly, variants within the gene locus encoding protein tyrosine phosphatase non-receptor type 2 (PTPN2) are strongly associated with increased risk for developing IBD, but also many other autoimmune/inflammatory diseases such as rheumatoid arthritis (RA) or type I diabetes (T1D) [4,5,21]. PTPN2 down-regulates pro-inflammatory cascades [22,23]. In fact, it has been shown that *Ptpn2* knock-out (KO) mice die spontaneously several weeks after birth due to a severe and systemic inflammation that affects multiple organs, including the gastrointestinal tract [24]. In the intestine, our group and others have demonstrated that PTPN2 protects the intestinal epithelial barrier function and regulates cytokine secretion by human monocytes and intestinal epithelial cells [23,25,26]. Moreover, the loss of *Ptpn2* in T-cells and myeloid cells resulted in more severe intestinal inflammation in mice [27,28].

Despite these data, limited information is available about the actual impact of TiO_2_ in IBD pathogenesis in a genetically susceptible host. To address this knowledge gap, we investigated the relationship between the food additive TiO_2_ and genetically caused *Ptpn2* dysfunction in myeloid cells during the course of DSS-induced colitis in vivo. We also studied the effects of TiO_2_ in vitro by using cultured bone marrow derived macrophages (BMDMs). We observed that TiO_2_ administration to mice lacking the expression of *Ptpn2* in myeloid cells (*Ptpn2^LysMCre^*) might exacerbate intestinal inflammation compared to their wild-type littermates, which could be most likely due to a shift in macrophage differentiation, impairing the development of anti-inflammatory M2 macrophages.

## 2. Results

### 2.1. TiO_2_ Induces a Different Intestinal Inflammation Profile in Mice Featuring Loss of Ptpn2 in Myeloid Cells 

**Figure 1:**
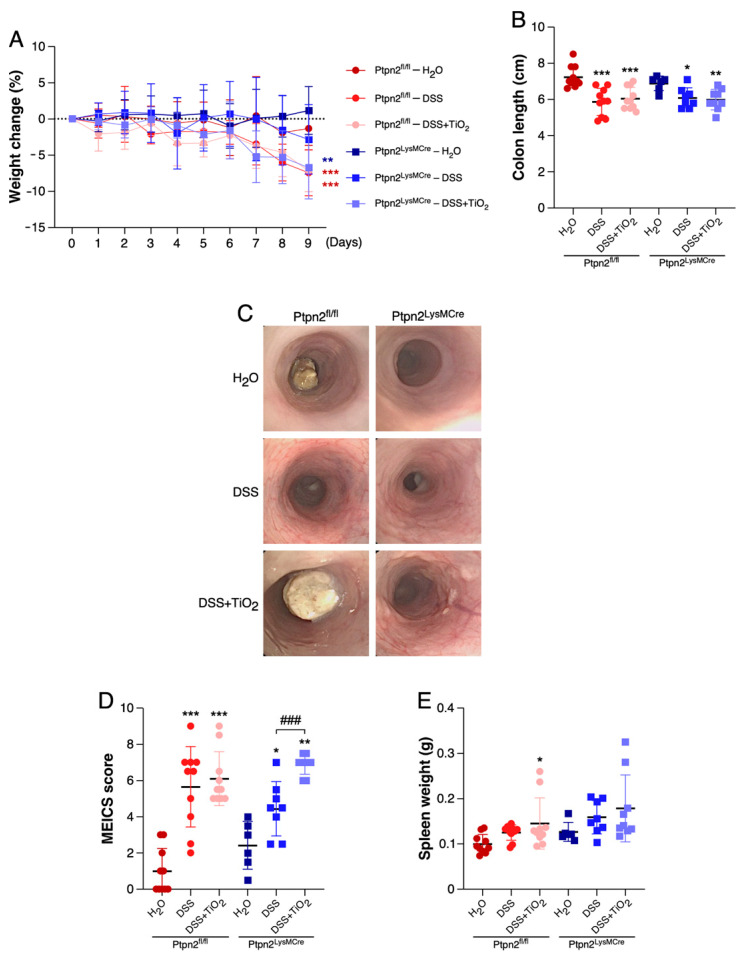
Loss of *Ptpn2* in myeloid cells exacerbates TiO_2_ effects at endoscopic level. (**A**) Percentage of weight change in *Ptpn2^fl/fl^* and *Ptpn2^LysMCre^* mice during DSS-acute colitis under the indicated treatments. (**B**) Representation of the colon length in *Ptpn2^fl/fl^* and *Ptpn2^LysMCre^* mice during DSS-acute colitis under the indicated treatments. (**C**) Representative endoscopic images of *Ptpn2^fl/fl^* and *Ptpn2^LysMCre^* mice during DSS-acute colitis under the indicated treatments. (**D**) Endoscopy scoring using the murine endoscopic index of colitis severity (MEICS) of *Ptpn2^fl/fl^* and *Ptpn2^LysMCre^* mice during DSS-acute colitis under the indicated treatments. (**E**) Representation of the spleen weight in *Ptpn2^fl/fl^* and *Ptpn2^LysMCre^* mice during DSS-acute colitis under the indicated treatments. Data shown in panels (**A,B,D,E**) represent mean ± SD. * *p* ≤ 0.05; ** *p* ≤ 0.01; *** *p* ≤ 0.001 relative to H_2_O of the same genotype. ^###^ *p* ≤ 0.001 relative to DSS of the same genotype.

In order to determine whether the impact of TiO_2_ administration on intestinal inflammation depends on the function of the IBD risk gene *Ptpn2* in myeloid cells, we induced acute colitis in *Ptpn2^LysMCre^* mice and *Ptpn2^fl/fl^* littermates. As shown in Figure 1A, while DSS treatment alone induced a clear weight reduction in either *Ptpn2^LysMCre^* or *Ptpn2^fl/fl^* mice, this effect was not significantly enhanced by co-administration of TiO_2_. Similar findings were obtained when analysing colon length as additional colitis read-out (Figure 1B). However, by mouse colonoscopy, we detected signs of more severe inflammation in the colon of DSS and TiO_2_ co-treated *Ptpn2^LysMCre^* when compared to DSS treated *Ptpn2^LysMCre^* mice. Accordingly, statistical analysis of the MEICS endoscopic score revealed a significantly higher score in TiO_2_ co-treated *Ptpn2^LysMCre^* mice in comparison to DSS-treated group of the same genotype, an effect that was not observed in *Ptpn2^fl/fl^* mice (Figure 1C,D). Spleen weight showed a tendency towards a slight increase in the TiO_2_ co-treated groups of both genotypes, compared to the spleen weight measured in DSS-treated groups (Figure 1E).

### 2.2. TiO_2_ Treatment Induces a Different Histologic Inflammation Profile in Mice Featuring Loss of Ptpn2 in Myeloid Cells

**Figure 2:**
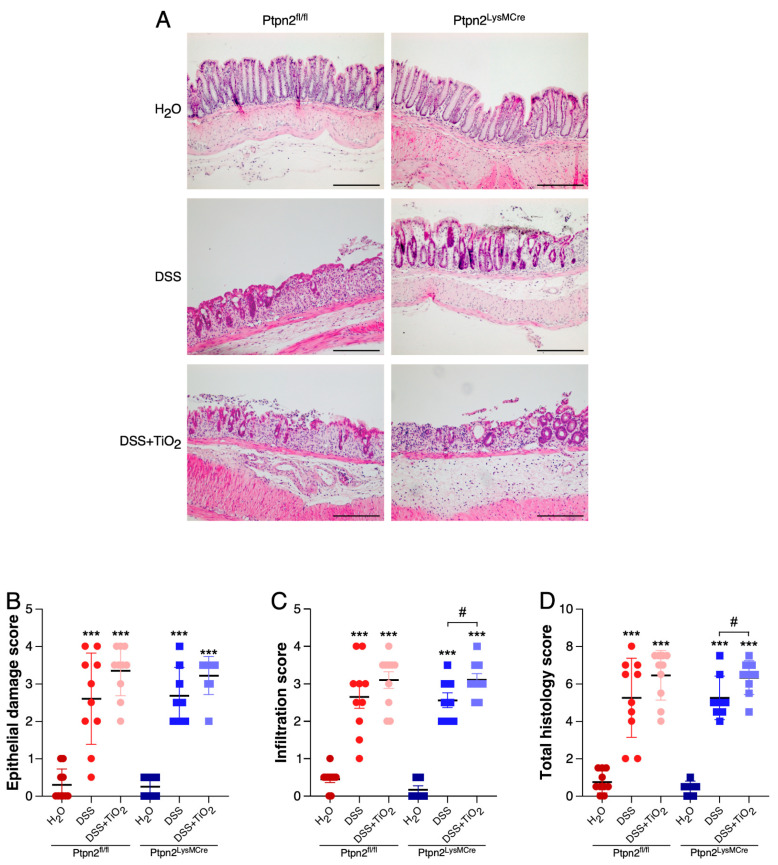
Loss of *Ptpn2* in myeloid cells exacerbates TiO_2_ effects at histological level. (**A**) Representative histology images of *Ptpn2^fl/fl^* and *Ptpn2^LysMCre^* mice during DSS-acute colitis under the indicated treatments. (**B**) Epithelial damage scoring in *Ptpn2^fl/fl^* and *Ptpn2^LysMCre^* mice during DSS-acute colitis under the indicated treatments. (**C**) Infiltration scoring in *Ptpn2^fl/fl^* and *Ptpn2^LysMCre^* mice during DSS-acute colitis under the indicated treatments. (**D**) Total histology scoring in *Ptpn2^fl/fl^* and *Ptpn2^LysMCre^* mice during DSS-acute colitis under the indicated treatments. Data shown in panels (**B**–**D**) represent mean ± SD. *** *p* ≤ 0.001 relative to H_2_O of the same genotype. ^#^ *p* ≤ 0.05 relative to DSS of the same genotype. Scale bar: 100 μm.

We next investigated whether the observed endoscopic differences were also detectable by histologic analysis of the mouse colon. As shown in Figure 2A, DSS induced damage of the epithelial barrier and infiltration of immune cells into the submucosa in mice of both genotypes. The administration of TiO_2_ in addition to DSS caused an even further increase in immune cell infiltration and a trend towards more severe epithelial damage. Accordingly, histological scoring revealed a strong increased tendency in the epithelial damage subscore and a significantly higher infiltration subscore and total histology score in *Ptpn2^LysMCre^* mice treated with TiO_2_ and DSS when compared to *Ptpn2^LysMCre^* mice treated with DSS alone. Of note, this effect was more pronounced in *Ptpn2^LysMCre^* mice than in their wild-type littermates (Figure 2B–D).

### 2.3. TiO_2_ Does Not Affect the Total Histological Numbers of T Cells and Macrophages or MPO Activity

**Figure 3:**
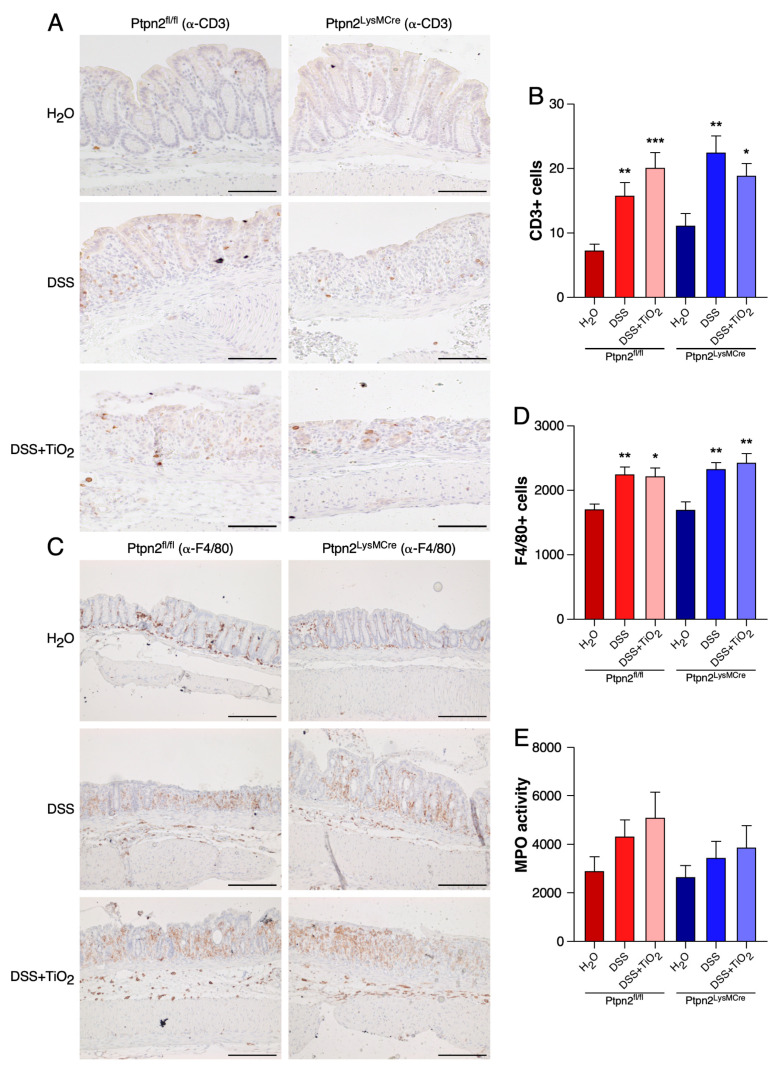
TiO_2_ does not modulate total numbers of T cells, macrophages and MPO activity. (**A**) Representative IHC images of the colon from *Ptpn2^fl/fl^* and *Ptpn2^LysMCre^* mice stained with anti-CD3 during DSS-acute colitis under the indicated treatments. (**B**) Quantification of the CD3 cells of IHC from *Ptpn2^fl/fl^* and *Ptpn2^LysMCre^* mice during DSS-acute colitis under the indicated treatments. C. Representative IHC images of the colon from *Ptpn2^fl/fl^* and *Ptpn2^LysMCre^* mice stained with anti-F4/80 during DSS-acute colitis under the indicated treatments. (**D**) Quantification of the F4/80 cells of IHC from *Ptpn2^fl/fl^* and *Ptpn2^LysMCre^* mice during DSS-acute colitis under the indicated treatments. (**E**) Representation of the MPO activity in *Ptpn2^fl/fl^* and *Ptpn2^LysMCre^* mice during DSS-acute colitis under the indicated treatments. Data shown in panels (**C**–**E**) represent mean ± SEM. * *p* ≤ 0.05; ** *p* ≤ 0.01; *** *p* ≤ 0.001 relative to H_2_O of the same genotype. Scale bar: 100 μm.

To gain insights into the in vivo mechanism explaining the differential TiO_2_ profiles during DSS-induced acute colitis in *Ptpn2^fl/fl^* and *Ptpn2^LysMCre^* mice, we analysed the expression of the pan-T cell marker CD3 (Figure 3A,B) and the macrophage marker F4/80 by IHC (Figure 3C,D). While DSS treatment caused a significant increase in the number of CD3 as well as of F4/80 positive cells in mice of both genotypes, this effect was not further enhanced by co-administration of TiO_2_. Also, as a measure of neutrophil infiltration, we performed an MPO assay. However, we did not detect any significant differences between treatment groups in either *Ptpn2^fl/fl^* mice or *Ptpn2^LysMCre^* mice (Figure 3E).

### 2.4. TiO_2_ Decreases the Number of Anti-Inflammatory Macrophages in LPLs

We next explored the composition of adaptive and innate immune cells by flow cytometry in LPL. These experiments allowed us to isolate specific populations, resulting in a more detailed study of the immune cell infiltration into the colon tissue during DSS-acute colitis. 

**Figure 4:**
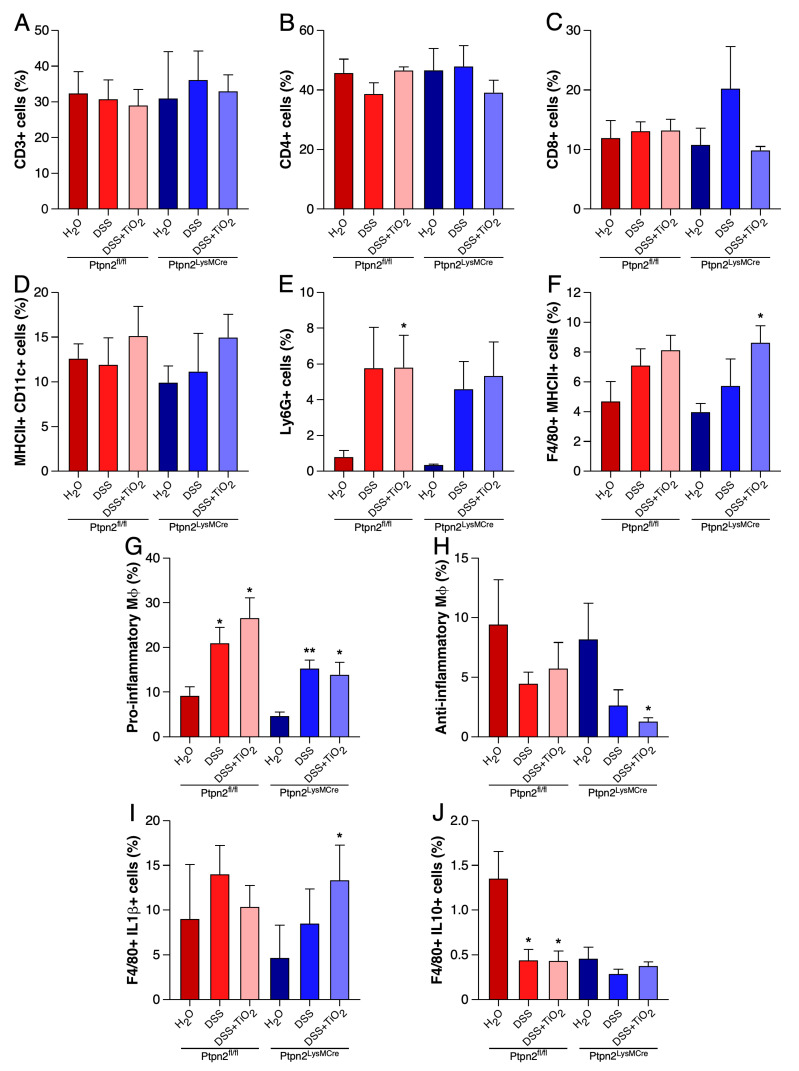
TiO_2_ down-regulates the frequencies of anti-inflammatory macrophage in LPLs. (**A**) CD3+ T cells detected by flow cytometry analysis in LPLs from *Ptpn2^fl/fl^* and *Ptpn2^LysMCre^* mice during DSS-acute colitis under the indicated treatments. (**B**) Frequencies of CD4+ T cells detected by flow cytometry analysis in LPLs from *Ptpn2^fl/fl^* and *Ptpn2^LysMCre^* mice during DSS-acute colitis under the indicated treatments. (**C**) Frequencies of CD8+ T cells detected by flow cytometry analysis in LPLs from *Ptpn2^fl/fl^* and *Ptpn2^LysMCre^* mice during DSS-acute colitis under the indicated treatments. (**D**) Frequencies of MHCII+ CD11c+ dendritic cells detected by flow cytometry analysis in LPLs from *Ptpn2^fl/fl^* and *Ptpn2^LysMCre^* mice during DSS-acute colitis under the indicated treatments. (**E**) Frequencies of Ly6G+ neutrophils detected by flow cytometry analysis in LPLs from *Ptpn2^fl/fl^* and *Ptpn2^LysMCre^* mice during DSS-acute colitis under the indicated treatments. (**F**) Frequencies of F4/80+ macrophages detected by flow cytometry analysis in LPLs from *Ptpn2^fl/fl^* and *Ptpn2^LysMCre^* mice during DSS-acute colitis under the indicated treatments. (**G**) Frequencies of pro-inflammatory macrophages (F4/80 gated; Ly6C+ MHCII+) detected by flow cytometry analysis in LPLs from *Ptpn2^fl/fl^* and *Ptpn2^LysMCre^* mice during DSS-acute colitis under the indicated treatments. (**H**) Frequencies of anti-inflammatory macrophages (F4/80 gated; Ly6C- MHCII+) detected by flow cytometry analysis in LPLs from *Ptpn2^fl/fl^* and *Ptpn2^LysMCre^* mice during DSS-acute colitis under the indicated treatments. (**I**) Frequencies of F4/80+ macrophages positive for IL-1β detected by flow cytometry analysis in LPLs from *Ptpn2^fl/fl^* and *Ptpn2^LysMCre^* mice during DSS-acute colitis under the indicated treatments. (**J**) Frequencies of F4/80+ macrophages positive for IL-10 detected by flow cytometry analysis in LPLs from *Ptpn2^fl/fl^* and *Ptpn2^LysMCre^* mice during DSS-acute colitis under the indicated treatments. Data shown in panels (**A**) to (**J**) represent mean ± SEM. * *p* ≤ 0.05; ** *p* ≤ 0.01 relative to H_2_O of the same genotype. Results from a representative experiment with four to five animals per group. Two independent experiments were performed.

As shown in Figure 4A, CD3+ T cell frequencies were similar in all animal groups and genotypes tested, confirming the observations made by the previous histology analysis. In the same way, CD4+ and CD8+ T cell subsets did not show any significant variation in the same conditions (Figure 4B,C). 

MHCII+CD11c+ dendritic cells presented similar frequencies among the different treatment groups and mouse genotypes (Figure 4D). Analysis of Ly6G+ neutrophils confirmed the results obtained with the MPO assay, showing an increased tendency in DSS- and DSS plus TiO_2_-treated groups, but with similar frequencies between genotypes (Figure 4E). F4/80 total and pro-inflammatory macrophages frequencies revealed a pattern similar to that observed for Ly6G+ cells or F4/80 in the IHC experiments (Figure 4F,G). Analysis of the subset of anti-inflammatory macrophages showed a trend towards a decrease in the numbers of this population in response to DSS treatment in mice derived from both genotypes. However, *Ptpn2^LysMCre^* mice treated with TiO_2_ revealed a further significant reduction in the number of anti-inflammatory macrophages when compared to controls of the same genotype, which was not observed in their *Ptpn2^fl/fl^* littermates (Figure 4H). Also, macrophages positive for IL-1β were significantly higher in *Ptpn2^LysMCre^* mice treated with TiO_2_ compared to the controls of the same genotype, but no difference was observed in *Ptpn2^fl/fl^* mice (Figure 4I). Finally, macrophages that were positive for the anti-inflammatory cytokine IL-10 were clearly reduced by DSS treatment in *Ptpn2^fl/fl^* mice. Of note, these cells were already very low in H_2_O and DSS treated *Ptpn2^LysMCre^* mice and DSS plus TiO_2_ treatment did not further reduce their numbers (Figure 4J).

### 2.5. TiO_2_ Induces IL-1β and Represses IL-10 Expression in BMDMs

**Figure 5:**
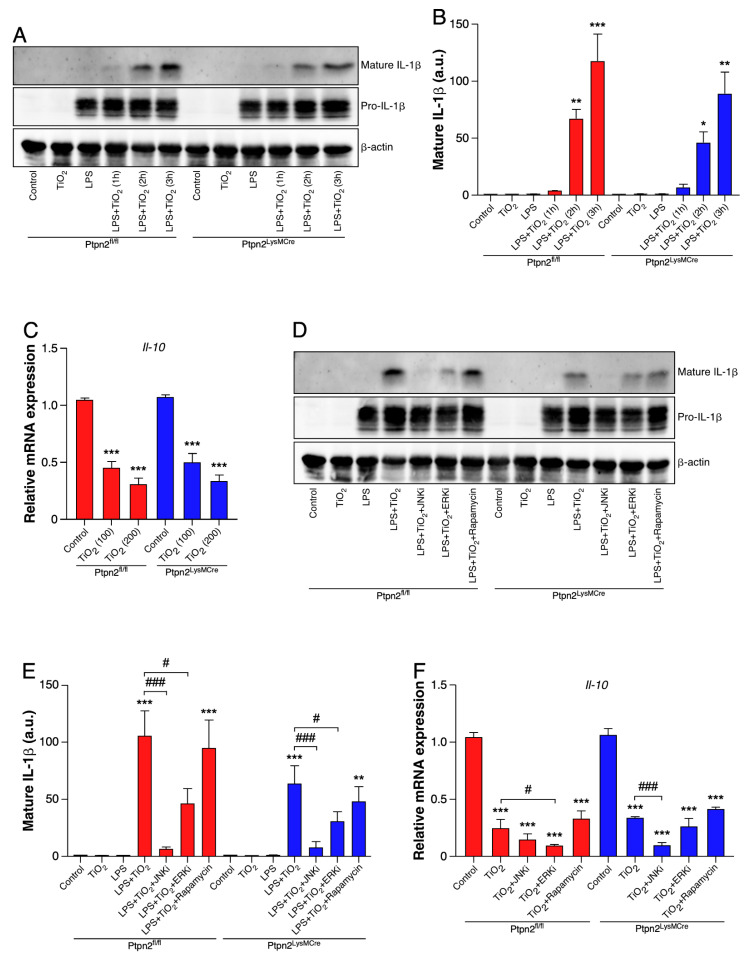
TiO_2_ up-regulates IL-1β and down-regulates IL-10 expression in BMDMs. (**A,B,D,E**), Representative Western blot images, showing the expression of the indicated proteins under the indicated treatments in BMDMs from *Ptpn2^fl/fl^* and *Ptpn2^LysMCre^* mice and the corresponding densitometric analysis expressed by arbitrary units (a.u.). (**C**,**F**) Levels of the indicated transcript under the indicated treatments in BMDMs from *Ptpn2^fl/fl^* and *Ptpn2^LysMCre^* mice. Data shown in panels (**B,C,E,F**) represent mean ± SEM. * *p* ≤ 0.05; ** *p* ≤ 0.01; *** *p* ≤ 0.001 relative to control of the same genotype. ^#^ *p* ≤ 0.05; ^###^ *p* ≤ 0.001 relative to LPS+TiO_2_ or TiO_2_ of the same genotype. Results from three independent experiments.

To confirm the involvement of TiO_2_ in the regulation of IL-1β and IL-10 in macrophages, we treated cultured BMDMs from *Ptpn2^fl/fl^* and *Ptpn2^LysMCre^* mice with TiO_2_. As shown in Figure 5A,B, while TiO_2_ treatment alone had no impact on IL-1β maturation, priming with LPS resulted in a clear, time-dependent increase in the levels of mature IL-1β in cell supernatant, in BMDMs from both genotypes. Moreover, the administration of TiO_2_ alone was already able to significantly repress the expression of IL-10 in the same cell type (Figure 5C). 

To gain further insights into the molecular mechanisms by which TiO_2_ regulates the expression of both cytokines, we used pharmacological inhibitors for different mitogen-activated proteins kinases (MAPKs), namely JNK and ERK, as well as for mammalian target of rapamycin complex 1 (mTORC1), which downstream effectors are much less dependent on MAPKs. As shown in Figure 5D,E, MAPK inhibition, especially JNK inhibition, abolished TiO_2_-induced secretion of mature IL-1β, in cell supernatant. In contrast, the mTORC1 inhibitor rapamycin did not have any significant effect in BMDMs of both genotypes. With respect to IL-10 mRNA levels, TiO_2_ again induced a clear decrease in IL-10 mRNA levels that was further potentiated in combination with MAPK inhibitors in BMDMs regardless of their genotype. On the other hand, mTORC1 inhibition had no impact on TiO_2_-induced inhibition of IL-10 mRNA expression (Figure 5F).

## 3. Discussion

In this study, we showed for the first time that Ptpn2 function in myeloid cells modulates the susceptibility to colon inflammation caused by the food additive TiO_2_. Using mice with a specific loss of *Ptpn2* in myeloid cells, we were able to demonstrate that TiO_2_ treatment shows different profiles in the extent of DSS-induced experimental colitis in *Ptpn2^LysMCre^* and *Ptpn2^fl/fl^* mice. Mechanistically, this effect seemed to be mediated by an altered macrophage polarisation. Particularly anti-inflammatory macrophages are significantly reduced in the intestine of *Ptpn2^LysMCre^* mice, an effect that was not observed in their wild type littermates.

In the last years, many studies have been highlighting the relevance and the detrimental effects of certain dietary components in the pathophysiology of a broad range of autoimmune, chronic inflammatory, metabolic and neurodegenerative diseases, such as IBD [10,29]. TiO_2_ is one of the inorganic microparticles most widely used by the food industry and has been related to the development of intestinal inflammation in pre-clinical models [11,14,16]. However, little is known about the molecular mechanisms by which TiO_2_ exerts its effects or whether different genetic backgrounds of the patients might increase the susceptibility to inflammation exerted by this inorganic compound. In the present study, we could not detect significant differences between genotypes after TiO_2_ treatment. However, we demonstrated that the loss of the IBD risk gene *Ptpn2* in myeloid cells produces a different pattern and a most robust tendency in the effects of TiO_2_ in the DSS acute model of experimental colitis.

Genetic ablation of *Ptpn2* has been found detrimental at the intestinal level. Lack of *Ptpn2* expression in either T cells or myeloid cells resulted in a more severe colitis [27,28] and knockdown of *PTPN2* in intestinal epithelial cells induced the expression of pro-inflammatory mediators [23]. However, here we provide the first evidence of the cooperation between the genetic factor *Ptpn2* and the food additive TiO_2_ in the promotion of intestinal inflammation. Similar interactions between industrial additives such as triclosan, a chemical used as an antimicrobial agent in toothpastes, cosmetics and other articles of daily life, and IBD risk genes were previously observed. Exposure to this compound increased the severity of DSS-induced colitis severity and this effect was abolished in *Tlr4* KO mice [30], suggesting that ingested inorganic molecules could use highly conserved components of the inflammatory response to impair the normal function of the immune system, and as a result, have an impact on intestinal homeostasis. 

With our present work, we additionally intended to isolate the cell type being responsible for the in vivo effects of TiO_2_. Unfortunately, a first approach by IHC did not reveal significant differences in the infiltration of T cell or macrophages in the intestinal lamina propria. Although IHC is a very useful technique, we were only able to study general T cell and macrophage populations with this method. Therefore, with this preliminary analysis some putative effects of TiO_2_ on specific immune cell sub-populations could be hidden. In fact, when we analysed specific T cell and macrophage populations by flow cytometry, we observed a reduction in the number of anti-inflammatory macrophages in the lamina propria of *Ptpn2^LysMCre^* mice, a decrease not detected in their wild-type littermates. In the case of neutrophils, the pattern observed by FACS confirmed the results obtained by MPO assay, which essentially is a measure of this peroxidase enzyme activity in neutrophils. TiO_2_ has been found cytotoxic for macrophages, increasing the apoptosis of RAW264.7 macrophage-like cell line [31]. In the same way, it has been reported that the administration of TiO_2_ microparticles to mice during DSS-induced chronic colitis reduced the number of macrophages in mesenteric lymph nodes [15]. To the best of our knowledge, this is the first study showing a reduction of anti-inflammatory macrophages in the lamina propria of mice lacking the expression of a member of the PTPN2 family in myeloid cells and treated with TiO_2_.

In order to gain more insights into the molecular mechanisms activated by TiO_2_ in macrophages, we analysed the production of the pro-inflammatory cytokine IL-1β and the anti-inflammatory mediator IL-10 in BMDMs. The observed induction in the secretion of mature IL-1β by TiO_2_ presented here is in agreement with an article previously published by our group, in which we demonstrated a similar effect in the THP-1 monocytic cell line [14]. On the other hand, we discovered, that this microparticle had an opposite effect on the expression of the anti-inflammatory cytokine IL-10, down-regulating this mRNA transcript in BMDMs. Both IL-1β and IL-10 are known as important regulators of intestinal inflammation and many studies demonstrate their participation in the onset and progression of IBD [32,33,34,35]. It is noteworthy that, conversely to what happened in vivo, the expression pattern of these cytokines is similar between genotypes in vitro. That could be explained by the complexity of the LPL microenvironment, which could have affected *Ptpn2^LysMCre^* macrophages phenotype in a different way in the animal setting as compared to the primary cell culture.

Finally, the signalling pathways by which TiO_2_ exerts its actions are poorly understood so far. For that reason, we aimed to identify putative signalling routes involved in TiO_2_ effects in vitro. We observed that the induction of mature IL-1β by TiO_2_ is mainly mediated via MAPK activity in BMDMs from both *Ptpn2^fl/fl^* and *Ptpn2^LysMCre^* mice, since the inhibition of pathways being independent on MAPKs activity, such as mTORC1 pathway, did not affect the secretion of mature IL-1β in response to TiO_2_ treatment. These results seem to be in line with observations made with other inflammasome activators such as monosodium urate (MSU) crystals [28]. However, the lack of a rescue effect in the expression of IL-10, using the same inhibitors as in the IL-1β experiments, suggests that TiO_2_ could act through different signalling pathways depending on whether this microparticle is promoting inflammation or repressing anti-inflammatory mediators.

In summary, our study demonstrates that the effects of the food additive TiO_2_ in the severity of intestinal inflammation could depend on the genetic background of the host. The loss of expression of the IBD risk gene *Ptpn2* in myeloid cells resulted in a different profile in the severity of colitis in mice exposed to TiO_2_. These results suggest that industrial compounds ingested in the diet should be carefully considered, with special attention in individuals with a genetic predisposition for IBD.

## 4. Materials and Methods

### 4.1. Animal Experiments

All mice used for the studies were homozygous for floxed *Ptpn2* gene and either heterozygous for the *LysMCre* construct (*Ptpn2^LysMCre^*) or without *LysMCre* (*Ptpn2^fl/fl^*). 10 to 12 weeks old female littermates housed in a specific pathogen-free facility (SPF) were used for the experiments. Pooled results from two independent experiments with four to five animals per group were shown.

Acute colitis was induced by the administration of 1.5% dextran sodium sulphate (MP Biomedicals, Carlsbad, CA, USA) in drinking water for 7 days. After that, DSS was replaced by normal drinking water for 2 more days. Treatment group was treated daily with a suspension of TiO_2_ (500 mg/kg/day) (IoLiTec, Heilbronn, Germany) in drinking water by oral gavage. TiO_2_ treatment started on the same day as DSS administration. On day 9, severity of colitis was assessed by endoscopy as previously described [14,36] and scored using the murine endoscopic index of colitis severity scoring system (MEICS). Briefly, MEICS score was calculated by an independent investigator blinded to the type of treatment. Endoscopic colitis score was based on the observed signs of inflammation by the analysis of different parameters: Thickening of the colon, changes in vascular pattern, fibrin visible, granularity of the mucosal surface and stool consistency. Each of these parameters was scored from 0 to 3 and the MEICS score was calculated by the sum of all of them. Animals were euthanized for sample collection. 

### 4.2. Histology

Colon tissue sections were fixed in 4% formalin, dehydrated by a graded series of ethyl alcohol (70 to 100%) and embedded in paraffin wax. 5 μm sections were cut using a rotary microtome (Zeiss, Oberkochen, Germany). For H&E staining, tissue sections were deparaffinized with Histo Clear (National Diagnostics, Atlanta, GA, USA), rehydrated using a graded series of ethyl alcohol (100 to 70%) and then stained for 10 min with hematoxylin (Schleicher&Schuell, Dassel, Germany) followed by a 2 s differentiating step with 1% HCL in ethanol and further stained for 10 sec with 1% eosin (pH 5.2). Finally, the sections were dehydrated with a series of ethyl alcohol (70 to 100%) and mounted with Pertex (HistoLab, Askim, Sweden).

For immunohistochemistry (IHC) staining, rehydrated samples were heated at 98 °C for 30 min in antigen retrieval solution pH 6.0 (Dako, Glostrup, Denmark). Endogenous peroxidases were blocked for 15 min with 0.9% H_2_O_2_ in PBS. Unspecific antibody binding was blocked by incubation with 3% BSA in PBS overnight, followed by primary antibody (Anti-CD3, Abcam, Cambridge, UK; anti-F4/80, Cell Signaling, Danvers, MA, USA) incubation at 4 °C for 1 h. After that, slides were incubated for 2 h with the secondary anti-rabbit antibody (Dako, Glostrup, Denmark) at room temperature followed by 1 min DAB staining (Dako, Glostrup, Denmark). Sections were counterstained for 10 sec with hematoxylin (Schleicher&Schuell, Dassel, Germany), dehydrated and mounted with Pertex (HistoLab, Askim, Sweden). The processed samples were examined under a light microscope Zeiss Axio Imager Z2 (Zeiss, Oberkochen, Germany) and images were taken at 10X magnification. Quantification of positive cells was performed using Image J Software version 1.53a.

### 4.3. Assessment of Histological Score

Histological scoring for inflammatory infiltration and epithelial damage was performed on hematoxylin and eosin (H&E) stained sections from the most distal part of the colon. Histological examination was performed by two independent investigators blinded to the type of treatment.

Epithelial damage: normal morphology = 0; loss of goblet cells = 1; loss of goblet cells in large areas = 2; loss of crypts = 3; loss of cryps in large areas = 4.

Infiltration: no infiltrate = 0; infiltrate around crypt basis = 1; infiltrate reaching to *L. muscularis mucosae* = 2; extensive infiltration reaching the L. muscularis mucosae and thickening of the mucosa with abundant oedema = 3; infiltration of the *L. submucosa* = 4.

The total histology score represents the sum of the epithelium damage score and the infiltration score.

### 4.4. Cell Culture

To generate bone marrow-derived macrophages (BMDMs), bone marrow was flushed from femurs and tibiae of *Ptpn2^fl/fl^* and *Ptpn2^LysMCre^* mice. After filtration and centrifugation, bone marrow cells were cultured in RPMI medium supplemented with 10% FCS and 10% L929 conditioned medium for 4 days. On day 4, the medium containing the non-adherent cells was removed and replaced with fresh medium for 2 more days. On day 6 fully differentiated BMDMs were obtained for subsequent experiments. Cells were detached after 10 min incubation with cold PBS plus EDTA 2 mM and seeded in p6 plates at 2 million cells per well density in RPMI medium supplemented with 10% FCS.

For inflammasome activation studies, BMDMs were starved overnight, primed with LPS (250 ng/mL) for 4 h and then treated with TiO_2_ (200 μg/mL) for 3 h, unless stated differently. Pharmacological inhibitors (see concentrations in the figure legend) were added 1 h before TiO_2_ stimulation.

For gene expression analysis, BMDMs were starved overnight and then treated with TiO_2_ (200 μg/mL) for 24 h, unless stated different. Pharmacological inhibitors (see concentrations in the figure legend) were added 1 h before TiO_2_ stimulation. 

### 4.5. Myeloperoxidase (MPO) Activity

Colon specimens (0.5 cm long) were homogenized in 50 mM phosphate buffer (pH 6.0) and 0.5% hexadecyltrimethylammonium bromide (Sigma-Aldrich, St. Louis, MO, USA) using a gentleMACS tissue homogenizer (Miltenyi Biotec, Bergisch Gladbach, Germany). After three freeze-and-thaw cycles, the supernatant was mixed with 0.02% dianisidine (Sigma-Aldrich, St. Louis, MO, USA) in 50 mM phosphate buffer, pH 6.0, and 0.0005% H_2_O_2_ (Sigma-Aldrich, St. Louis, MO, USA). MPO activity, expressed as arbitrary units, was calculated as mean absorbance (460 nm) per incubation time (in minutes) per protein content (in grams).

### 4.6. Flow Cytometry

Lamina propria lymphocytes (LPLs) were used for flow cytometry analysis. Single-cell suspension from LPLs was prepared by cutting a colon segment in approximately 0.5 mm^3^ pieces and then incubated with HBSS plus EDTA 2 mM for 15 min at 37 °C in a shaker. After washing with HBSS, colon pieces were incubated in HBSS plus EDTA 2 mM buffer for 30 min at 37 °C in a shaker. Then, colon pieces were digested using a digestion buffer containing dispase (0.6 mg/mL) and collagenase IV (0.4 mg/mL) for 20 min at 37 °C in a shaker. Digested colon pieces were finally homogenized by using a syringe and 18G needle (BD, USA) and filtered through a 70 μm cell strainer (BD, Franklin Lakes, NJ, USA).

LPL cell suspensions were re-stimulated with PMA (50 ng/mL) (Sigma, USA), Ionomycin (1 μg/mL) (Sigma-Aldrich, St. Louis, MO, USA) and Brefeldin A (5 μg/mL) (Sigma-Aldrich, St. Louis, MO, USA) for 4 h. Cells were stained using the following fluorescent-labelled antibodies for 20 min: BV510-labelled anti-CD45 (BioLegend, San Diego, CA, USA); APC or PE-Cy5.5-labelled anti-CD3 (BioLegend, San Diego, CA, USA); PE-Cy5-labelled anti-F4/80 (BioLegend, San Diego, CA, USA); AF700-labelled anti-MHCII (BioLegend, San Diego, CA, USA); PE-Cy7-labelled anti-CD11c (BioLegend, San Diego, CA, USA); BV711-labelled anti-Ly6C (BioLegend, San Diego, CA, USA); PE-labelled anti-Ly6G (BioLegend, San Diego, CA, USA); APC-labelled anti-IL-1β (Invitrogen, Carlsbad, CA, USA); BV605-labelled anti-IL10 (BioLegend, San Diego, CA, USA); APC-Cy7-labelled Zombie NIR (BioLegend, San Diego, CA, USA) was used to stain dead cells.

For intracellular cytokine staining, cells were fixed and permeabilized using the BD Cytofix/Cytoperm buffer (BD, Franklin Lakes, NJ, USA) for 20 min, followed by fluorescent-labelled antibodies staining for 20 min. Cells were analyzed on an LSR Fortessa cytometer (BD, Franklin Lakes, NJ, USA).

### 4.7. Western Blot

Whole cell protein extraction was developed using M-PER Mammalian Protein Extraction Reagent (Thermo Fisher Scientific, Waltham, MA, USA), following manufacturer´s instructions. For isolation of mature secreted IL-1β, cell culture supernatant was mixed with acetone (2.5 times the supernatant volume) and incubated overnight at −20 °C. On the next day, the supernatant plus acetone mixture was centrifuged at 14.000 g, 50 min, 4 °C. The obtained pellet was washed once with an acetone 90% dilution in milliQ H_2_O, centrifuged 14.000g, 5 min, 4 °C and resuspended in M-PER lysis buffer (Thermo Fisher Scientific, Waltham, MA, USA).

Lysates were separated by SDS-PAGE on a 10% or 12% polyacrylamide gel. Proteins were subsequently transferred to a nitrocellulose membrane. Membranes were blocked, primary antibodies were incubated overnight and the appropriate HRP-conjugated secondary antibody was applied for 1 h. Proteins were detected by using the Chemiluminescence Reagent for Horseradish Peroxidase (Witec AG, Sursee, Switzerland) and the Fusion Solo S imager (Vilber, Collégien, France) using anti-IL-1β antibody (R&D Systems, Minneapolis, MN, USA) as well as anti- β-actin antibody (Merck-Millipore, Burlington, MA, USA) as loading control.

### 4.8. RNA Isolation and RT-qPCR 

mRNA levels were determined using TaqMan technology. Briefly, total RNA was extracted using the Maxwell RSC simply RNA Tissue Kit (Promega, Madison, WI, USA) according to the manufacturer´s instructions. Reverse transcription was performed using the High-Capacity cDNA Reverse Transcription Kit (Thermo Fisher Scientific, Waltham, MA, USA) following the manufacturer´s instructions. After the RT reaction, real-time PCR was performed using the TaqMan Fast Universal PCR Master Mix and specific TaqMan expression assays for mouse IL10 and mouse β-actin (endogenous control). All measurements were done in triplicate using the QuantStudio 6 Flex System (Thermo Fisher Scientific, Waltham, MA, USA) according to the supplier´s instructions and the results were obtained by the comparative ΔΔCt method.

## 5. Statistics

If not otherwise stated, Student’s *t*-test was applied. Sample size and the number of independent experiments for each experiment are indicated in the appropriate figure legend. Experimental values in graphs are provided as mean and SEM, unless stated differently. Results with *p* values ≤ 0.05 were considered statistically significant.

## Data Availability

The data presented in this study are available on request from the corresponding author.

## Abbreviations

DSS	Dextran Sodium Sulfate
IBD	Inflammatory Bowel Disease
TiO_2_	Titanium dioxide
PTPN2	Protein tyrosine phosptahatse non-receptor type 2
LPL	Lamina propria lymphocytes
BMDMs	Bone marrow-derived macrophages
MAPK	Mitogen-activated protein kinase
IL	Interleukin
JNK–c-JUN	N-terminal kinase
ERK	Extracellular signal-regulated kinase
mTORC1	Mammalian target of rapamycin complex 1
Tlr4	Toll like receptor 4
